# Supplementary material for: Effect of holiday admission for acute aortic dissection on in-hospital mortality in Japan: A nationwide study
Source: PLoS One. 2021 Nov 18;16(11):e0260152. doi: 10.1371/journal.pone.0260152 (PMC8601417; doi:10.1371/journal.pone.0260152)
Supplement: S1 Table — (DOCX) [file pone.0260152.s001.docx]

**S1 Table. Characteristics of patients with acute aortic dissection according to sex.**

|  | Men | Women | p-value |
| --- | --- | --- | --- |
| Number | 14,730 | 10,911 |  |
| Age^a^ | 67 ± 13 | 75 ± 12 | < 0.001 |
| Current smoking (%)^b^ | 10,633 (72.2) | 3,319 (30.4) | < 0.001 |
| Comorbidity |  |  |  |
| Hypertension | 10,423 (70.8) | 6,923 (63.4) | < 0.001 |
| Diabetes mellitus | 1,421 (9.6) | 923 (8.5) | 0.001 |
| Dyslipidemia | 2,724 (18.5) | 1,813 (16.6) | < 0.001 |
| Heart failure | 2,681 (18.2) | 2,137 (19.6) | 0.005 |
| Myocardial infarction | 550 (3.7) | 336 (3.1) | 0.005 |
| Cerebrovascular disease | 1,130 (7.7) | 1,086 (10.0) | < 0.001 |
| Renal disease | 815 (5.5) | 399 (3.7) | < 0.001 |
| Cancer | 569 (3.9) | 271 (2.5) | < 0.001 |
| Stanford type A | 5,869 (39.8) | 6,929 (63.5) | < 0.001 |
| Surgery (%)^b^ | 4,291 (29.1) | 4,038 (37.0) | < 0.001 |
| Endovascular | 487 (11.3) | 150 (3.7) | < 0.001 |
| Open Surgery | 3,804 (88.7) | 3,888 (96.3) |  |
| Hospitalization days | 25 ± 20 | 25 ± 22 | 0.94 |
| In-hospital mortality (%)^b^ | 1,886 (12.8) | 2,225 (20.4) | < 0.001 |

^a, b^ Data are expressed as mean ± standard deviation, or number (%).
