# Supplementary material for: Effect of holiday admission for acute aortic dissection on in-hospital mortality in Japan: A nationwide study
Source: PLoS One. 2021 Nov 18;16(11):e0260152. doi: 10.1371/journal.pone.0260152 (PMC8601417; doi:10.1371/journal.pone.0260152)
Supplement: Attachment — Submitted filename: responsetothereviewers20210902.docx [file pone.0260152.s002.docx]

**RESPONSE TO THE REVIEWERS**

On behalf of all the authors, I would like to thank the Reviewers for their valuable suggestions, which have helped us to revise and improve our manuscript. I would also like to thank the Editor for the kind remarks.

**RESPONSE TO REVIEWER:**

**Reviewer #1:**

1. **The abstract should be divided in the appropriate sections.**

Thank you for pointing this out. We have divided the Abstract sections: Background, Methods, Results, and Conclusion.

2. **The method of categorization of patients used to perform the analysis was described in the section “Data definition”. It should be moved in the section “Study setting and patients selection”.**

Thank you for pointing this out. We have moved these sentences to the “Study setting and patients selection” section.

3. **In the section “Measurement”, it was described only the primary outcome of the study. This item should be improved.**

Based on your comment, we have added information on the secondary outcome and variables that may be controversial, such as in-hospital mortality.

4. **The information regarding the patient’s selection and the definition of population analysed in the study, in association with the flow-chart of the patient selection process, were reported in the section “Results”. It should be moved in the “Methods” section.**

Based on your comment, we have moved the said sentences to the “patients’ selection process” in the Methods.

5. **The Table 1 and Table 2 showed the characteristics of the patients according to sex and to Stanford type. The variables analysed are few. In particular, many pre-operative variables, including cardiovascular risk factors, that could help to understand the risk profile of patients are not analysed.**

Thank you for pointing this out. We have added data of patients’ comorbidities (i.e. hypertension, diabetes, dyslipidemia, congestive heart failure, myocardial infarction, cerebrovascular disease, renal disease, and cancer).

6. **In Table 2, they reported that 8065 patients (23.9%) of the overall population were “Unknown”. This group of patients should be removed from the analysis.**

We have removed the “Unknown” group of Stanford type patients from the analysis. As a result, we reanalyzed our data and reported the results accordingly.

7. **In Table 2, the authors reported that 58.2% of acute type A aortic dissection were treated surgically. How were the remaining patients treated?**

Within this database, we could not know the detailed treatment progression of each patient. The remaining patients were medically treated.

8. **The type of aortic surgery performed is not reported.**

We have added the type of surgical intervention (endovascular or open surgery) in the Tables. Open surgery was performed in 98.8% of the Stanford type A group. On the other hand, endovascular repair was performed in 62.7% of the Stanford type B group.

9. **The post-operative complications of patients underwent aortic arch surgery should be added.**

Unfortunately, this database does not contain information about post-operative complications. We mentioned this as one of the study limitations (lack of postoperative date in the database).

10. **The authors reported in the limitation of the study the lack of the pre-operative, intra-operative and post-operative information, but these data are important to comprehend the risk profile of the patients and the results of the study.**

We acknowledge the importance of this information in this study. However, the database does not contain information about post-operative complications.

11. **The Table 3 reported the logistic regression analysis for in-hospital mortality for weekday, Saturday, Sunday/holiday and for every 7 days of the week. The two different analysis are reported in two different tables that should be numbered separately. Moreover, in these tables the results are difficult to understand.**

Based on your comment, we have separated these two tables (Table 3 and Table 4).

12. **Among acute type B aortic dissection, both surgically and medically treated patients were included. These two types of patient population could not be considered in the same group. The patients with acute type B aortic dissection surgically treated have anatomic and hemodynamic instability requiring urgent surgical/endovascular treatment. Instead, patients medically treated are stable and had lower risk of morbidity and mortality.**

Based on your suggestion, we divided the patients into the four groups (Stanford type A and surgical intervention (-), Stanford type A and surgical intervention (+), Stanford type B and surgical intervention (-), and Stanford type B and surgical intervention (+)) in the stratified analysis. In the present analysis, only 6.8% of patients with Stanford type B had surgical intervention. In the stratified analysis, in-hospital mortality of patients with Stanford type B admitted on　Sunday/holiday in both the surgical intervention (-) and (+) group was not significantly different from those admitted during the weekdays.

13. **In order to find a correlation between admission day and in-hospital mortality, it may be more interesting to analyse in detail the single population of patients admitted in the hospital for acute type A aortic dissection and treated with emergent surgical operation. In fact, as the authors of this study also report, the acute Type A aortic dissection seems more vulnerable to “weekend effect” than the acute Type B aortic dissection medically treated.**

Based on your suggestion, we divided patients with Stanford type A according to presence or absence of surgical intervention. In the stratified analysis, patients with Stanford type A and presence of surgical intervention seemed more vulnerable to the “weekend effect” than absence of surgical intervention.

**Reviewer #2:**

**Methods**

14. **As far as I can tell, you have used ANOVA for comparing weekdays vs weekends.　Have you performed ad hoc analysis in order to determine where the significant results peak? Or pairwise testing with P-value correction.**

Given that this study did not aim at knowing the differences between the particular days, we did not perform an ad hoc analysis. This study rather focused on investigating the difference between the presence or absence.

15. **You use frequently stratified analysis term: make describe it under statistical analysis**

Thank you for pointing this out. We have improved the statistical analysis section.

**Results**

16. **Table 2 lacks a statistical test between Type A and B.**

Based on your comment, we added the statistical test used in comparing between Type A and B groups.

17. **In both logistic regression analysis your weekdays vs sunday/holiday is containing saturday?**

Saturday was included in both logistic analyses.

18. **In stratified analysis p-value and CI reporting there is discordance: if CI contains 1 results can not be significant, please do revisit your analysis**

Based on your suggestion, we performed a re-analysis and provided the CIs where available, to justify the significant differences.

19. **From table make clear comparing groups with its p values**

Thank you for pointing this out. We have included the p values in the tables and added more information on the statistical test used in the Method.

20. **I truly and honestly think there is something else, only week day can not explain the complexity of the AAD mortality. It could be interesting to understand time of admission (morning shift or evening shift), shortage of professionals, ect. Please do share your depersonalized data.**

As your righty commented, the factors influening AAD mortality are complex. The weekend effect involves several factors; however, our database did not contain the exact time of admission.

**Reviewer #3:**

21. **Aortic dissection type-A and type B have very different severities and they are very distinct disease entities: most A need big open surgery and most B need only endovascular or non-surgical treatments. They should not be mixed in the same article. If it does, please make sure to discuss them separately.**

Thank you for pointing this out. We stratified our data according to the presence/absence of surgical intervention and presented the results accordingly. Furthermore, we discussed the findings in the Discussion section.

22. **Here the grouping was defined as "admission date". Please specify in more detail. Was it referred to actual clock time or admin time? Was your date starting from 8:01 am or 0:01 am? For example, was 1 am on Monday classified as Monday or Sunday? By admin time, Monday starts from 8am Monday but manpower at 1 am Monday still belongs to Sunday. Please clarify. This confusion will cause a huge misclassification.**

Thank you for pointing this out. The time range for admissions in our study was 00:00 am–11:59 pm. 　The patients’ real admission time reflected the admission day.

23. **Here in the Title "weekends" always include Saturdays. But in your manuscript, Saturdays were not treated the same as Sunday or holidays. If your major grouping was Sundays/holidays vs weekdays/non-holidays, please revise your Title accordingly--it's not "weekend" effect! Revised to "holiday" effect or maybe some other better term. Please clarify.**

Thank you for pointing this out. We have changed the title from “weekend admission” to “holiday admission.”

24. **What are the non-weekend holidays in Japan? Please specify. Also, was there any holidays celebrated differently in different parts in Japan--namely, was there any holiday off in some parts but non-off in other parts in Japan? In this nationwide study, this geographical holiday variations must be considered if any.**

Non-weekend holidays (i.e., national holidays) are same in all parts of Japan. We have added information in the methods to clarify this issue.

25. **The outcome of "in-hospital mortality" needs more clarification. In Asian culture, some family prefer the patients to die at home What if the dying patient, alive when leaving hospital, was transferred home to die naturally. Was it "in-hospital mortality"? Also, how about the OHCA cases? If they were resuscitated and able to be diagnosed as aortic dissection, were they included or excluded in the study cohort? If these OHCA aortic dissection cases were supported by ECMO throughout the course until withdrawal, were they (dead before arrival to hospital) considered "in-hospital death"? There are some confusions requiring clarification in the manuscript. Please address them properly.**

Thank you for pointing these out. Patients who were in a dying state upon discharge (left to die naturally at home) or patients supported by ECMO throughout the course until withdrawal were not considered as “in-hospital mortality.” It is true that, some patients and their families in Japan prefer to die at home, however, very few patients in dying state with emergency conditions were left to go home. Furthermore, our database contained data of patients who were brought to the hospital for cardiac arrest state and were diagnosed with AAD. Such patients’ deaths were considered in-hospital mortality.

**Reviewer #4:**

26. **Difference between groups (weekend and weekday) are missing and the final results should be adjuasted for differences. Table 1 reports difference according to gender which is not the issue under evaluation. Without a correct adjustment no conclusions can be traced**

We have changed Table 1 from the difference between men and women to difference between weekend and weekday groups. The table according to sex has been considered as Supplementary Table1.

**POINTS OF REVISION**

All changes are noted in red in the text.

**･TITLE PAGE:**

·P. 1 L. 3: We corrected the text in accordance with the reviewer’s suggestion.

·P. 1 L. 16, 17: We corrected the text in accordance with the English proofreading.

**･ABSTRUCT:**

·P. 2 L. 22, 28, 36, P3 L. 45: We add text in accordance with the reviewer’s suggestion.

·P. 2 L. 37, 38, 40, 41, P3 L. 43-47: We changed the text because results of statistical test had changed.

·P. 3 L. 49, 50: We corrected the text in accordance with the English proofreading.

**･INTRODUCTION:**

·P. 4 L.56, 60, 71: We corrected the text in accordance with the English proofreading.

**･METHODS:**

·P. 5 L. 95: We corrected the text in accordance with the English proofreading.

·P. 6 L. 103-120, P. 7 126-134: We transferred and added text in accordance with the reviewer’s suggestion.

·P. 7 L. 136-141: We added the text in accordance with the reviewer’s suggestion.

·P. 8 L. 146–148, 149-150, 157-158: We added the text in accordance with the reviewer’s suggestion.

**･RESULTS:**

·P. 8 L. 164-165, P. 9 L. 166-170: We deleted text in accordance with the reviewer’s suggestion.

·P. 9 L. 172-173: We added the text in accordance with the reviewer’s suggestion.

·P. 9 L. 174, 176, 177, 178, 179: We changed the text because results of statistical test had changed.

·P. 9 L. 182-183: We changed the text in accordance with replace the tables.

·P. 11 L. 187-188: We added the text in accordance with replace the tables.

·P. 14 L. 191: We added the text in accordance with added the Supplementary table1.

·P. 15 L. 196: We added the text in accordance with the reviewer’s suggestion.

·P. 15 L. 200, 201: We corrected the text in accordance with the English proofreading.

·P. 15 L. 208, 209, 211: We changed the text because results of statistical test had changed.

·P. 16 L. 212-213, 215-221: We changed the text because results of statistical test had changed.

·P. 16 L. 223-224: We changed the text in accordance with reviewer’s suggestion.

·P. 19 L. 228, P. 20 L. 232: We added the text in accordance with reviewer’s suggestion.

·P. 20 L. 230, 231, 233, P. 21 L. 234: We corrected the text in accordance with the English proofreading.

**･DISCUSSION:**

·P. 21 L. 239, 243, 252, 255, 257, 258, 260, 261, 263, 266-267, 272, 273, 275, 279, P. 22 L. 281, 282, 283, 291, 297: We corrected the text in accordance with the English proofreading.

·P. 22 L. 276-277: We added text in accordance with the reviewer’s suggestion

**･CONCLUSION:**

·None

**･REFFERENCES:**

·None

**･TABLES:**

· We changed the Table 1 and Table 2 in accordance with re reviewer’s suggestion.

·We added the Supplementary table1.

·We separated Table 3 for Table 3 and Table 4. In accordance with reviewer’s suggestion.

**･TABLES:**

·In Figure 2, we changed the text in accordance with the study population.
