# Supplementary material for: Effect of holiday admission for acute aortic dissection on in-hospital mortality in Japan: A nationwide study
Source: PLoS One. 2021 Nov 18;16(11):e0260152. doi: 10.1371/journal.pone.0260152 (PMC8601417; doi:10.1371/journal.pone.0260152)
Supplement: Attachment — Submitted filename: responsetothereviewers20211026.docx [file pone.0260152.s003.docx]

**RESPONSE TO THE REVIEWERS**

On behalf of all the authors, I would like to thank the Reviewers for their valuable suggestions, which have helped us to revise and improve our manuscript. I would also like to thank the Editor for the kind remarks.

**RESPONSE TO REVIEWER:**

Reviewer #2: Methods

Not clear what stands for surgical intervention (-) or (+), make clearer statements.

Response: Thank you for raising this point. We have revised the　descriptions to ensure clarity and avoid any confusion.

Results

In all tables in caption or footnote the specific test has been used for comparison.

In Figure 2 try to use different types of plot, for example boxplot! And provide an ad hoc test for better weekday separation and significance.

Response: Thank you for your suggestion. We have changed Figure 2 to a boxplot and performed an additional ad hoc test.

**POINTS OF REVISION**

All changes are noted in red in the text.

**･TITLE PAGE:**

·None

**･ABSTRUCT:**

·P. 3 L. 44-46: We changed the text in accordance with the reviewer’s suggestion.

**･INTRODUCTION:**

·P. 4 L.57: We corrected the text in accordance with the English proofreading.

·P. 4 L.77: We corrected the text in accordance with the English proofreading.

**･METHODS:**

·P. 6 L. 102-103: We changed the text in accordance with the reviewer’s suggestion.

·P. 6 L. 99, 104, 105: We corrected the text in accordance with the English proofreading.

·P. 7 L. 143-144, P. 8 L. 145: We changed the text in accordance with the reviewer’s suggestion.

·P. 8 L. 148-149, 152: We added the text in accordance with the reviewer’s suggestion.

**･RESULTS:**

·P. 8 L. 160, 162: We corrected the text in accordance with the English proofreading.

·P. 8 L. 165-166: We changed the text in accordance with the reviewer’s suggestion.

·P. 8 L. 169-170, P. 10 L. 173-174, P. 11 L. 177: We corrected the text in accordance with the English proofreading.

·P. 12 L. 172-173: We changed the text because results of statistical test had changed.

·P. 13 L. 188-189: We changed the text because post hoc test statistical test had added.

·P. 13 L. 193, 198-199, 202-203: We changed the text in accordance with the reviewer’s suggestion.

·P. 13 L. 207, P. 14 L. 210: We corrected the text in accordance with the English proofreading.

**･DISCUSSION:**

·P. 17 L. 240, P. 18 L. 242, P. 19 L. 279: We corrected the text in accordance with the English proofreading.

·P. 18 L. 258-259: We changed the text in accordance with the reviewer’s suggestion

**･CONCLUSION:**

·None

**･REFFERENCES:**

·None

**･TABLES:**

· We changed the Table 1, Table 2, S1Table, Table 3, and Table 4 in accordance with re reviewer’s suggestion.

**･FIGURES:**

·In Figure 2, we have changed to a boxplot.
